# The Influence of the Mandibular Chin Angle on the Occurrence of Mandibular Condylar Fracture: A Retrospective Study

**DOI:** 10.1155/2021/2380840

**Published:** 2021-12-20

**Authors:** Sunil S. Nayak, S. Arun, Abhay Taranath Kamath, Bharath Jaladhigere Lakshmanagowda, Eshita Dubey, Jonathan Koshy

**Affiliations:** ^1^Department of Oral & Maxillofacial Surgery, Manipal College of Dental Sciences, Manipal Academy of Higher Education, Manipal, Karnataka, India; ^2^Department of Oral & Maxillofacial Surgery, KMC Hospital, Mangalore, India; ^3^Department of Radio Diagnosis and Imaging, Kasturba Medical College, Manipal Academy of Higher Education, Manipal, Karnataka, India

## Abstract

**Background:**

Condylar fractures are commonly associated with symphysis/parasymphysis fractures. Condylar fractures have been attributed to direct and indirect traumatic forces, the direction and magnitude of the forces, and the condylar anatomy. The chief aim of this study was to determine the association between the newly defined mandibular chin angle and the occurrence of condylar fractures.

**Materials and Methods:**

A retrospective study was conducted to analyze two-dimensional computed tomography (2D CT) scans of patients with a history of chin trauma. The outcome was a symphysis/parasymphysis fracture with or without fracture of the mandibular condyle. The Mediff InstaRISPACS web-based platform was used to measure the chin angle. The cerebral aqueduct of Sylvius in the corresponding 2D CT midsagittal image was the standard reference plane to measure the chin angle. The SPSS Version 20 (IBM Corp, Armonk, NY) was used for data analysis.

**Results:**

The sample size included 120 2D CT scans of patients with symphysis/parasymphysis fractures (60 associated with condylar fractures and 60 without condylar fractures). The mean chin angle in the group without condylar fracture was 133.35 ± 3.87°, which was approximately 15° lesser than in the condylar fracture group (mean, 148.56 ± 5.49°), and these findings were statistically significant (*P* < 0.05).

**Conclusion:**

Individuals with a high chin angle are potentially at a higher risk of sustaining associated condylar fractures.

## 1. Introduction

The mandible, being the most prominent bone in the facial skeleton, is vulnerable to trauma. Of all facial fractures, mandibular fractures range from 12% to 56% [1, 2]. 29% to 52% of these mandibular fractures are condylar fractures [3, 4]. The mandibular fracture location strongly correlates with age, sex, dental trauma, and soft-tissue injury. The mandibular anatomy, bone mineral density, and masticatory muscles are internal factors that influence fracture patterns [5]. The magnitude and direction of the external forces acting on the mandible dictate the fracture pattern and the location of fractures [6]. Finite-element analysis studies of mandibular biomechanics have identified potential weak areas in the mandibular structure based on the distribution of tensile and compressive stresses [7, 8].

Sympyseal fractures are significantly associated with condylar fractures [9]. Whenever there is an anterior blow to the mandible, the area of impact acts like a lever resulting in condylar fractures [10]. The condylar fractures due to a parasymphyseal impact are caused by the unequal distribution of mechanical stress in the mandible. The variable stress distribution is correlated with anterior mandibular morphological characteristics [11, 12]. The chin position, prominence, and anterior mandible height could be considered factors in determining the resistance of the symphyseal/parasymphyseal anatomy. These factors also determine the influence of an impact on the anterior mandible on indirect condylar fractures [13, 14]. A new angle called the chin angle is defined in the present study to assess the influence of the anterior mandibular morphology on the occurrence of condylar fractures. The Chin angle can be digitally measured in the midsagittal section of the 2-dimensional computed tomography (2D CT) scan. This study aims to determine any possible correlation between mandibular chin morphology (chin angle) and mandibular condylar fractures.

## 2. Materials and Methods

In this retrospective study, the data of patients who were surgically treated for fractures of the mandible from August 2019 to December 2020 were collected. Approval from the institutional review board was obtained before the commencement of the study (IEC: 523/2019).

The inclusion criteria comprised preoperative computed tomographic (CT) scans of patients who reported to the hospital with a history of chin trauma involving a symphysis/parasymphysis fracture with or without fracture of the mandibular condyle. CT scans of patients with a pathological fracture of the mandible, those with mandibular asymmetry, and malposed anterior teeth formed the exclusion criteria. CT scans and reports of a total of 120 patients with a symphysis/parasymphysis fracture (60 with condylar fracture and 60 without condylar fractures) following trauma to the chin region, satisfying the inclusion criteria, were evaluated.

**Figure 1:**
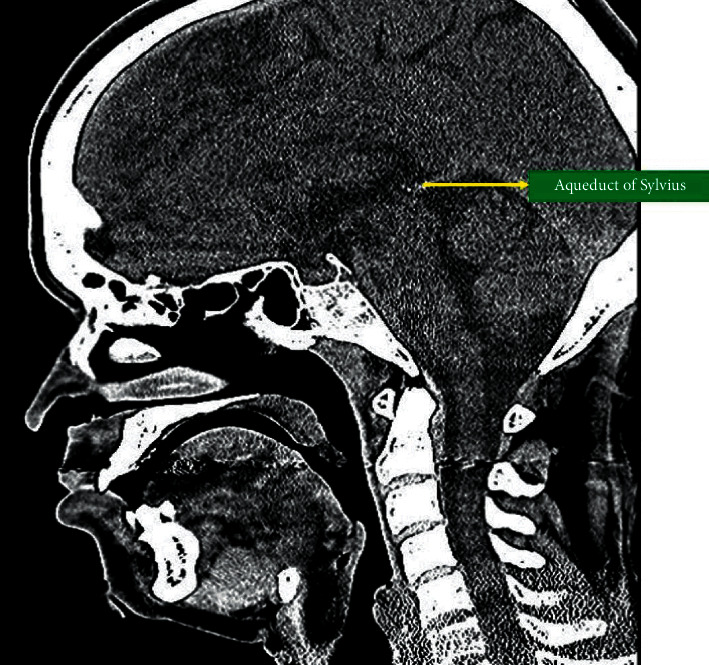
The aqueduct of Sylvius in the 2D midsagittal CT image.

The CT images of the patients were captured using the “Philips Incisive CT 128 Slice” machine, which uses “Dual Flying Focus Technology” as per the manufacturer's specifications. The CT scan data were then archived on the Mediff InstaRISPACS web-based platform specifically customized and designed for our hospital. The 2D CT images were accessed from the same for this study purpose. The 2D midsagittal CT image with the cerebral aqueduct of Sylvius as the anatomic reference plane was used to measure the chin angle (Figure 1).

The study sample was broadly classified into the following:

- Symphysis/parasymphysis fracture with associated condylar fractures
- Symphysis/parasymphysis fracture without any associated condylar fractures

### 2.1. Method of Chin Angle Measurement

**Figure 2:**
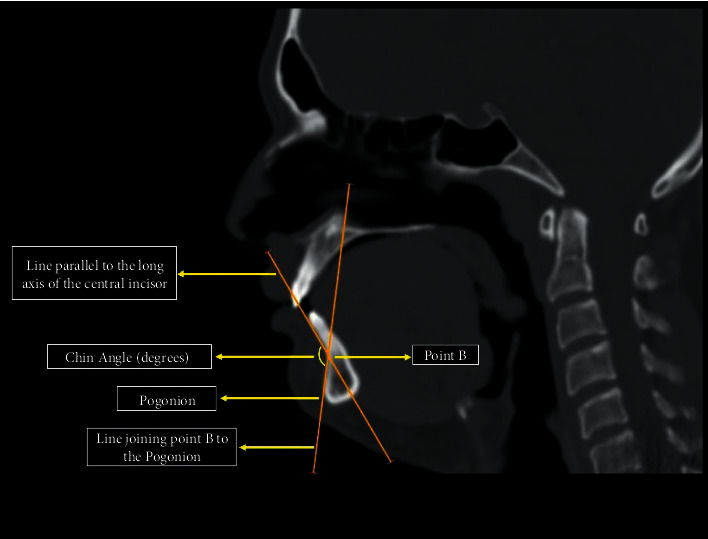
The chin angle depicted in the midsagittal CT scan.

In the 2D midsagittal section of the CT scan, the cerebral aqueduct of Sylvius was identified. The corresponding chin angle was measured in the same midsagittal plane. This newly defined angle is formed by the intersection of a line parallel to the long axis of the central incisor passing through the root apex and the line joining point *B* (the deepest point on the anterior border of the mandible) to the point pogonion (most anterior point on the mandible symphysis) (Figure 2).

Levene's test for equality of variances was used to compare quantitative variables. A *P* value less than 0.05 was considered statistically significant for the study. SPSS Version 20 (IBM Corp, Armonk, NY) was used for data analysis.

## 3. Results

The sample size included 120 2D CT scans of patients with symphysis/parasymphysis fractures (60 associated with condylar fractures and 60 without condylar fractures). The group without condylar fractures included 10 women (16.66%) and 50 men (83.33%), whereas the group with condylar fractures included 11 women (18.33%) and 49 men (81.66%). The mean age distribution in patients with condylar fractures was 29.92 ± 11.6 and was 33.35 ± 11.31 in the noncondylar fracture group.

**Figure 3:**
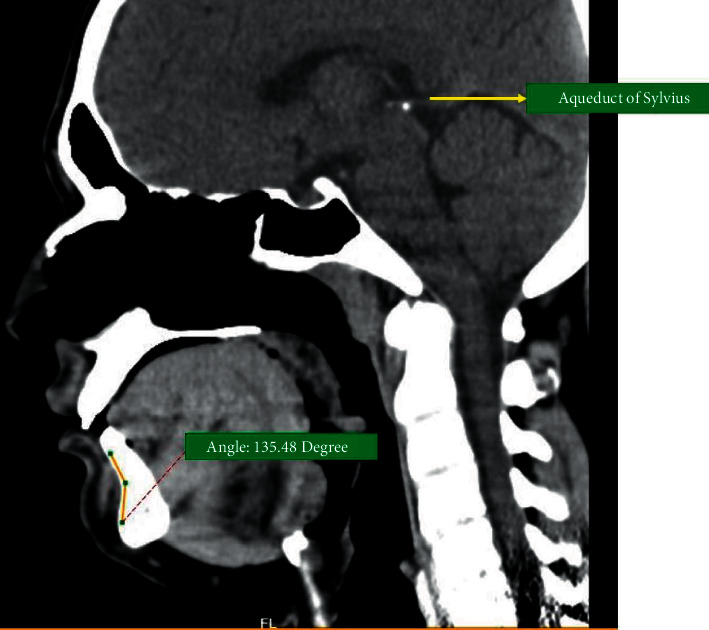
Chin angle in a 2D CT scan of a noncondylar fracture patient.

**Figure 4:**
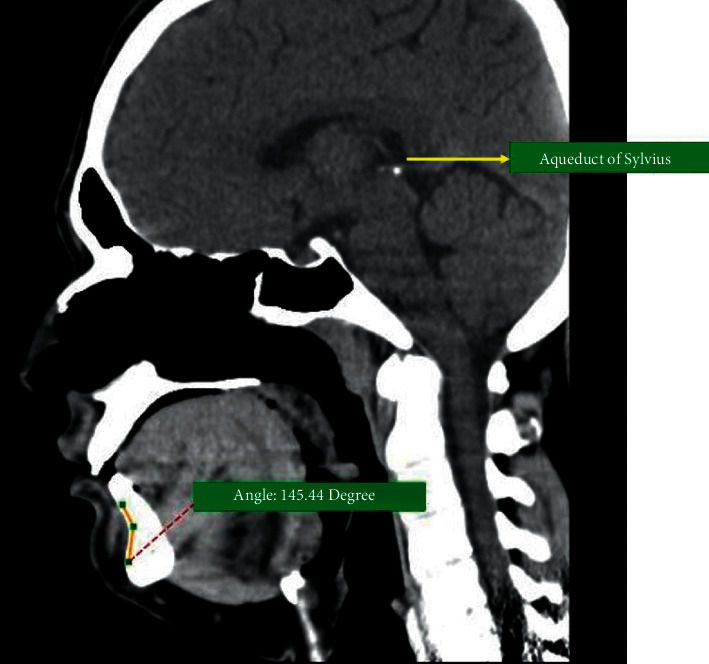
Chin angle in a 2D CT scan of a condylar fracture patient.

**Figure 5:**
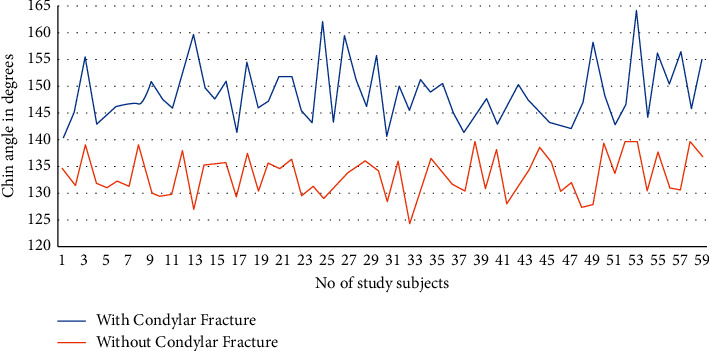
Comparison of chin angles in condylar fracture and noncondylar fracture groups.

The mean chin angle in the group without condylar fracture was 133.35 ± 3.87° (Figure 3), which was approximately 15° lesser than in the condylar fracture group (mean, 148.56 ± 5.49°) (Figure 4). This observation was statistically significant according to Levene's test (*P*=0.041; Table 1). The analysis of data indicates a significant association between the chin angle and condylar fractures. The association of condylar fractures with high chin angle cases was significantly increased (Figure 5).

## 4. Discussion

Fracture of the mandibular condyle is frequently encountered in oral and maxillofacial surgical practice. The occurrence of condylar fractures has a multifactorial etiology. Several studies have reported various factors for the occurrence of condylar fractures, such as weaker areas along the length of the mandible, the effect of direct and indirect forces of trauma acting on the mandible, and the condylar morphologic features. The forces of trauma are usually distributed along the entire length of the mandible [15]. However, various ridges, curvatures, and reduced cross-sectional areas (subcondylar region) contribute to its uneven structure [16–18]. This uneven structure of the mandible results in some areas which are weaker than others. The weakest point in the mandibular arch is usually affected by the forces applied and can cause tensile failure and extreme bending in that region [18]. The bending of the mandibular neck region can lead to tension failure and manifest as condylar fractures.

According to a review by Zachariades et al., 72% of condylar fractures are related and exist along with other mandibular fractures such as parasymphyseal fractures [19]. When trauma occurs, a direct fracture can be seen on the impact site, and an indirect fracture can be seen contralaterally [15]. According to Hulke et al., a force applied to the symphysis region causes widening of the lingual cortex and chin flattening resulting in a symphyseal fracture due to the tensile strain produced [20]. The bony glenoid fossa and soft tissues in the region tend to limit the mobility of the condylar process as it moves away from the point of impact [21]. When the mandible is subjected to a greater force externally in the anterior region, a symphysis fracture with an associated condylar fracture (unilateral/bilateral) occurs [22]. Various studies have shown that unilateral or bilateral condylar fractures are due to high energy impact and excessive force during mandibular trauma [9, 19, 22]. Moreover, the site and size of the direct fracture site and the surface area of the impact determine the indirect fracture site [15].

Internal factors such as bone mineral density, mandibular anatomy, and masticatory muscles can also affect the fracture pattern. According to Han et al., the morphological features of the mandibular chin region correlate with condylar fractures occurring due to an impact in the parasymphyseal area [5]. The distribution of stress in the mandibular ramus and body region varies due to an impact in the parasymphysis region and can result in a condylar fracture. A change in the ramus morphology may affect the transmission of forces [5]. Similarly, in the present study, the authors suggest changes in the chin morphology (symphyseal and parasympyseal region) affect forces transmitted to the condyles. These forces can influence the occurrence or nonoccurrence of condylar fractures. This study shows a correlation between high chin angle and the occurrence of condylar fractures associated with a symphysis/parasymphysis fracture and is found to be statistically significant. The symphysis/parasymphysis cases without an associated condylar fracture had a comparatively low chin angle. According to Xin et al., condylar head fractures due to an impact on the parasymphysis region correlate with the condylar anatomy. The present study shows that the anatomical variations in the symphyseal (chin) region can also influence condylar fractures [10].

A computed tomography (CT) scan will provide a complete injury assessment of facial fractures, particularly in cases of high-energy impact or severe comminution [23, 24]. The 2D midsagittal image is an information-rich image within multiplanar imaging sets of the brain. A large number of anatomic structures and spaces can be identified on midsagittal images of the brain. The cerebral aqueduct of Sylvius connects the third ventricle to the fourth ventricle and can be viewed in the midsagittal image of the brain CT. This narrow channel ranges from 1 to 3 mm in diameter [25]. In the present study, the chin angle was measured in this particular midsagittal image with the cerebral aqueduct of Sylvius as the fixed reference plane to eliminate any observer bias. The correlation of the chin angle and the occurrence of condylar fractures were determined on this basis.

Pannerselvam et al. highlighted decreased bone stock and cortical bone thickness in the mandibular angle region in high-gonial-angle cases [26]. The risk of mandibular angle fracture in patients with high gonial angles was significantly more [26, 27]. Similarly, the present study results show that a higher chin angle can be a possible risk factor in condylar fractures and can be attributed to the decreased bone density in the symphyseal region. More significant displacement of the fracture fragments is possible in high chin angle cases with associated symphysis/parasymphysis and condylar fractures. Various approaches and dissection techniques have been advocated for the surgical management of condylar fractures. The risk of facial nerve injury is not influenced by the location of the incision but is significantly influenced by the route of dissection [28]. The position and height of the condylar fracture determine the surgical approach. The miniretromandibular approach is an ideal approach to manage condylar fractures at all levels. This approach employs the transmasseteric anteroparotid route and can be performed easily with no damage to the facial nerve [29].

There are certain limitations to this study. The amount of external force acting on the symphysis/parasymphysis region due to trauma was a variable that could not be assessed in this study. A multicentric study with larger sample size and in vitro finite-element analysis could provide more information in this regard.

## 5. Conclusions

This study showed that individuals with a high chin angle are potentially at a higher risk of sustaining associated condylar fractures.

## Figures and Tables

**Table 1:** Summary of outcome variables.

Group	Range (degrees)	Mean chin angle (degrees)
Without condylar fracture	124.4–139.88	133.35 ± 3.87
With condylar fracture	140.51–164.71	148.56 ± 5.49
Significance (*P* value)	0.041	

## Data Availability

The data used to support the findings of this study are available from the corresponding author upon request.
